# Multidisciplinary cancer care in Spain, or when the function creates the organ: qualitative interview study

**DOI:** 10.1186/1471-2458-11-141

**Published:** 2011-02-28

**Authors:** Joan Prades, Josep M Borràs

**Affiliations:** 1 Catalan Cancer Plan, Hospital Duran i Reynals, 199-203 Avenue Gran Via de l'Hospitalet, Hospitalet de Llobregat 08908, Spain; 2 Clinical Sciences Department, Institut de Recerca Biomèdica de Bellvitge (IDIBELL) - Universitat de Barcelona, 199-203 Avenue Gran Via de l'Hospitalet, Hospitalet de Llobregat 08908, Spain

## Abstract

**Background:**

The Spanish National Health System recognised multidisciplinary care as a health priority in 2006, when a national strategy for promoting quality in cancer care was first published. This institutional effort is being implemented on a co-operative basis within the context of Spain's decentralised health care system, so a high degree of variability is to be expected. This study was aimed to explore the views of professionals working with multidisciplinary cancer teams and identify which barriers to effective team work should be considered to ensure implementation of health policy.

**Methods:**

Qualitative interview study with semi-structured, one-to-one interviews. Data were examined inductively, using content analysis to generate categories and an explanatory framework. 39 professionals performing their tasks, wholly or in part, in different multidisciplinary cancer teams were interviewed. The breakdown of participants' medical specialisations was as follows: medical oncologists (n = 10); radiation oncologists (n = 8); surgeons (n = 7); pathologists or radiologists (n = 6); oncology nurses (n = 5); and others (n = 3).

**Results:**

Teams could be classified into three models of professional co-operation in multidisciplinary cancer care, namely, advisory committee, formal co-adaptation and integrated care process. The following barriers to implementation were posed: existence of different gateways for the same patient profile; variability in development and use of clinical protocols and guidelines; role of the hospital executive board; outcomes assessment; and the recording and documenting of clinical decisions in a multidisciplinary team setting. All these play a key role in the development of cancer teams and their ability to improve quality of care.

**Conclusion:**

Cancer team development results from an specific adaptation to the hospital environment. Nevertheless, health policy plays an important role in promoting an organisational approach that changes the way in which professionals develop their clinical practice.

## Background

The increasing complexity of cancer care makes organisation of clinical decision-making one of the key elements in high-quality cancer care [1]. This raises the question of how good professional co-operation is to be achieved in day-to-day clinical practice. Pre-eminent among the different answers to this question is multidisciplinary teamwork, an approach that has emerged in parallel with the accelerated process of specialisation in cancer among health professionals. Recent reviews of the literature have associated a multidisciplinary approach to cancer care with better adherence to clinical practice guidelines [2], increased patient access to clinical trials [3] and enhanced co-ordination of hospital services [4,5]. These outcomes, together with the role assigned to multidisciplinary care in various cancer plans, indicate its strategic role for health systems in general and for quality of care in particular in the organisation of cancer services, something that was highlighted at the Lisbon round-table held under the Portuguese EU Presidency (2007) [6].

The development of multidisciplinary cancer care involves a redistribution of health professionals' tasks when it comes to clinical decision-making and patient care processes. The development of specific organisational frameworks for dealing with different types of cancer care must be seen in the context of a services-based hospital structure. This process modifies a highly sensitive aspect, i.e., the way in which professionals interact and are co-ordinated. The following are the main aspects defining a multidisciplinary approach in the organisation of cancer care:

- professional specialisation by disease (including diagnostic disciplines);

- standardisation of the process and clinical criteria in guidelines and pathways;

- redistribution of tasks at a multidisciplinary-team level; and,

- trend towards identifying and allocating specific resources according to disease/organ

The Spanish National Health System (SNHS) recognised multidisciplinary care as being a health priority as far back as 2006, when a national strategy for promoting quality in cancer care was first published [7]. As one of its basic principles, this *Cancer Strategy *document stipulates that cancer patients should be diagnosed and treated in the context of a multidisciplinary team (MDT), and goes on to identify tumour boards as the main mechanism for deciding and planning therapy. This institutional effort is being implemented on a co-operative basis within the context of Spain's decentralised health care system. The priority assigned by the respective regional health services to multidisciplinary care since health service management became operationally decentralised (2002) and the specific mode of organisation introduced at each hospital, together determine the starting point of the implementation of an MDT approach. A high degree of variability is thus to be expected.

This study addresses the question of how multidisciplinary cancer care has been implemented and the critical factors linked to this process, with special stress laid on the knowledge of policy required to ensure effective team work. The study was undertaken against a common backdrop of a growing cancer care burden and a rapidly expanding range of potentially effective treatments, which involves "therapeutic dilemmas about treatment options" [8]. A qualitative approach was chosen in order to better understand the perceptions and beliefs of all the professional partners involved at the different public hospitals spread across the nation's various health care regions.

## Methods

### Study design and setting

A qualitative research method was used in order to describe health professionals' points of view and experiences of multidisciplinary cancer care, and explore the barriers to be considered in future policy development. As an MDT approach in cancer had not been previously studied in the context of the Spanish health system, a pilot test was deemed appropriate. The aim was to identify a set of analytical categories which, along with a review of the literature, would define a theoretical basis for the interviews. The pilot scheme was undertaken by three teams at different hospitals in Catalonia, focusing on different tumours (breast, lung and colorectal). In order to ensure the relevance and appropriateness of the categories yielded by the test, health professionals from different disciplines were then asked to give their considered opinion. The methodology of analysis consisted of semi-structured interviews conducted *in situ *from October 2008 to January 2009 with professionals involved in the diagnosis and treatment of cancer patients at public hospitals of differing levels of importance, situated in the most populated regions of Spain, namely, Andalusia, Catalonia, Madrid, Galicia and Valencia. Participants were interviewed by an experienced, qualitative researcher.

### Recruitment

A total of thirty-nine health professionals were recruited. They were deemed eligible in any case where they performed their professional task, wholly or in part, in an MDT. For the selection of informants and composition of the theoretical sample, three inclusion criteria were established. To ensure that the views of different health professionals could be explored, the first criterion laid down that five medical specialisations had to be covered in each region: these were medical oncology, radiation oncology, surgery, pathology or radiology, and oncology nursing (Table 1). The second criterion reinforced a systematic approach to the phenomenon under study, by insisting on the presence of three opinions from each region (table 2). The third criterion took the form of a restriction on interviewing professionals belonging to the same team, with the aim of preventing biased versions on the subject of study and so contribute to the internal validity of our research.

**Table 1:** Detailed breakdown of the 39 professionals interviewed

Medical specialisations:	No. of professionals
	

Medical oncologist	10

Radiation oncologist	8

Surgeon	7

Radiologist/Pathologist	6

Nurse	5

Other (palliative care, gynaecologist)	3

	

Cancer site:	

Breast	19

Colorectal	8

Lung	4

Other	8

**Table 2:** Profiles required for selection of key informants

From a process standpoint	Professionals usually working with this type of organisational approach
From a technical standpoint	Professionals who have led organisational change towards more integrated forms of multidisciplinary cancer care

From an institutional standpoint	Professionals who frame the situation of multidisciplinary care within a hospital management model

### Interviews

A semi-structured list of questions ensured that critical points were covered in every interview. To elicit beliefs and experiences, participants were given the necessary flexibility to enable them to volunteer information on topics that were relevant to them. The selected health professionals were interviewed on a one-to-one basis for 60-100 minutes at their hospital offices. Interviews started with a general question on the cancer team's approach and ended with a question on how multidisciplinary care could be promoted by health policy measures. No notes were taken by the researcher during the interview; instead, all interviews were audio-taped and transcribed in full by the researcher. These data were then compiled into a documentary record and rendered anonymous to protect confidentiality. Every transcription was checked against its corresponding audio record and accuracy was found to be good. A preliminary analysis was conducted after each interview.

### Analysis

Interview data were examined inductively, using content analysis to generate categories and an explanatory framework. Grounded theory methodology was considered appropriate for describing the organisation and culture of health professionals belonging to multidisciplinary cancer teams. As our study was theoretical and aimed at incorporating the organisational context in which cancer teams practised, we used an axial coding, as described by Strauss and Corbin [9]. Data were electronically coded with ATLAS.ti [10]. Whereas the thematic analysis enabled language use to be understood and professionals' beliefs to be communicated, the method of constant comparison ensured that recurring views and experiences were obtained. The coding process and emerging themes were derived, on the one hand, from *a priori *issues drawn from the pilot test and previous research, and on the other, from issues raised by participants. Examples of codes were "nature of agreements", "access of a patient to the team during his/her journey" and "impact management on care performance". The consistency of coding/interpretation was checked during analysis by reviewing the transcripts at different moments in time. This process allowed for labelling and developing a reference of the data for subsequent exploration and identification. Accordingly, a thematic framework based on models of and barriers to effective multidisciplinary teamwork was identified. A specific effort was made to capture this stage of interpretation, i.e., by mapping, creating typologies [11] and finding associations among themes. Moreover, the preliminary findings were discussed at a workshop (held by the scientific societies), to which some of the health professionals interviewed and a number of social science researchers were invited. These discussions were useful for reinforcing team types for the Spanish health system and clarifying some barrier-related aspects.

### Ethical considerations

The data for this study was based on professionally conducted interviews which, other than the consent of the professionals themselves, require no "ethical approval" from any research committee. However, as the aim of our study is sensitive to hospital organisation and relations between specialisations and health professionals, confidentiality is of the essence. Accordingly, the strategy pursued was to prioritise a selection of participants whose opinion on these issues was deemed crucial, and so, contact with regional cancer control policymakers was made. The implementation of the study began through contact with the heads of the regional cancer plans, who proposed a short list of health professionals selected in accordance with our criteria (see table 2). Likewise, the resulting list was endorsed by the scientific societies of medical and radiation oncology. The health professionals concerned were then sent a letter of invitation explaining the research goals and a confidentiality agreement. On being advised by telephone and receiving an assurance as to the confidentiality of any information provided, all the professionals selected for study consented to the interviews being recorded. The consent form was formally signed at the meeting, with any doubts as to the designated purpose and method of research being discussed with the professionals. No-one refused the invitation to participate.

## Results

We identified three models of development of multidisciplinary cancer care into which all teams could be classified (table 3). Their internal consistency means that they can be seen as *models of co-operation*, and despite being general categories, any given team model may well contain elements of the other two. Moreover, the qualitative classification described here may even occur within the same hospital for different teams, with each of these assuming responsibility for a different tumour.

**Table 3:** Models of co-operation in multidisciplinary cancer care

	1. Advisory committee	2. Formal co-adaptation	3. Integrated care process
Cases submitted (approx. %)	"Complex" cases or off-protocol: 10% - 50%	All "possible" cases: 50% - 80%	Initial source of clinical assessment: 90%-100%

Patient access to team	Treatment (whether or not initiated)	Diagnosis or treatment	Suspect or diagnosis *(early access)*

Nature of agreements	Recommendations	Consensus decisions not always implemented	Binding decisions defended by the team

Professional team roles	Negative perception	Chair, tumour board co-ordinator	Chair, co-ordinator, nurse case manager

Impact on clinical process management	Minor changes	Some segments of care	Whole process (*cross-boundary *frequent)

Specialist participation	No diagnostic specialisations	Absences due only to timetable problems	Professionals associated with a clinical committee

Junior doctors and nursing role, in terms of attendance	Considered inappropriate	Open meeting, participation encouraged	Mandatory presence

Hospital executive board role	Lack of interest	Acknowledgement without express support	Express support (room, clerk, etc.)

Presence in health system	40%	50%	10%

Rather than targeting specific forms of organisation (tumour board, cancer unit), this approach focuses instead on team capabilities, based on each team's work method and the overall scope (breadth and depth) of the tasks performed, elements of analysis that emerged during the study and indicate the nature of interaction among professionals. While all cancer teams fulfilled the role of assessing patients and complying with and updating clinical protocols, obvious differences in their respective abilities to achieve quality of care were nevertheless observed.

### 1.- Advisory committee

This is a group of professionals, largely made up of specialists in different therapeutic fields, which meets regularly on a informal basis to discuss cases considered clinically complex. Since the patients may already have received some of the treatments (usually surgery), the multidisciplinary meeting is aimed at referring them to other professionals for further treatment. This approach implies rigorous respect for the autonomy of the clinician and for overlapping boundaries between the team and the multidisciplinary meeting: patient assessment is made without other health care performance considerations. Judging by our results, this model continues to enjoy an important presence in the system (40%).

### 2.- Formal co-adaptation

Owing to the high degree of interaction (mutual adaptation) among the professionals involved, consensus plays a key role in this model. The team acts as the reference framework for professionals, who share their views on the diagnosis, treatment and monitoring of a specific cancer type. The meeting is open to all professionals involved in patient management and it is here that the roles of team tumour board co-ordinator and chair appear. The key factor for fostering such an approach is agreement on the need for: joint decision-making to precede application of any treatment; and all cases to be dealt with at the multidisciplinary meeting. Both aspects are hampered, however, by hospital service inertia when it comes to disease management. This formula accounts for half (50%) of all MDT meetings in the health care system.

### 3.- Integrated care process

The teams that work under this model share wide aims on patient management, including co-ordination of clinical research and economic evaluation of treatment. As this model provides teams with early access to patients, the latter's preferences along with knowledge of their co-morbidities and psychosocial context are incorporated into the multidisciplinary discussions. This occurs through systematic follow-up of patients throughout their journey, from suspicion of cancer, to diagnosis, therapeutic decision-making and follow-up. The presence of professional team roles has an impact on the entire care process and on progress towards achieving seamless care. The hospital executive board plays a pivotal role by consolidating meeting times, and ensuring health professionals' attendance as well as their commitment to the MDT. This model is not frequent in the system (10%).

### Critical factors in multidisciplinary cancer team development

For many clinicians, development of multidisciplinary care has involved a cultural change, as can be seen from the pathway which resulted from *recommendations *that were proposed by cancer team members for clinical management of patients and have gradually become *binding decisions*. The key critical factors identified for this change are as follows:

#### Existence of different gateways for the same patient profile

Many clinicians acknowledged significant variability in clinical practice as a result of diagnosing and treating patients who, despite having similar symptoms and diagnoses, might receive different initial therapy because access to hospital took place through different departments. A typical example is provided by the different therapeutic approaches proposed to a prostate cancer patient depending on the initial hospital department responsible for his diagnosis. Although a shared clinical protocol on such patients for the whole hospital plus an agreement to submit the patient to the MDT meeting would limit this variability, it is the unification of hospital-access gateways into a single hospital department that would have the necessary transforming quality in terms of standardisation of clinical criteria and pathways. Indeed, a recurring example of this type of organisational change is afforded by unification of admission to radiology for breast cancer or to gastroenterology for colorectal cancer in the case of patients displaying symptoms with a high risk of cancer. The experience of agreeing upon a common gateway for suspects has three effects: it provides teams with early access to patients; it reduces the feeling of patients being the "property" of any given clinician or department; and it sets up a primary care reference catchment area, as it becomes easier and clearer to determine where and how subjects with high risk of cancer should be referred and who the specialists of reference are. Moreover, where the department taking on the gateway unification process of the clinical pathway is a diagnostic unit, this implies that it should have a more relevant role within the team.

"At the hospital, there is a breast cancer unit that is beset by a root problem, i.e., two treatment options depending on whether the patient has been admitted via gynaecology or surgery. These are internal battles waged by the respective competencies." (Breast surgeon)

"In this hospital there are two chairs of surgery: one comes to the meetings, the other doesn't. We know that they administer different forms of treatment. The percentage of cases in which this occurs is by no means inconsiderable." (Medical oncologist)

#### Variability in the development and use of clinical protocols and guidelines

Evidence-based decisions are a source of concern to professionals, and the updating of clinical protocols by the team reflects this concern. Many clinicians felt, however, that this goal was conditioned by the implementation and dissemination of cancer clinical guidelines in the Spanish Health System. They argued that the absence of common guidelines for the whole country and the lack of co-ordination strategies for implementing the few that did exist resulted in reduced use and a lack of systematic assessment of existing levels of adherence. Owing to this perceived situation, clinical protocols at a hospital level are very often based on foreign guidelines, and efforts made to produce Spanish ones are of little use. This in turn leads to three common situations which impact at a team level. Firstly, hospitals that refer cases (e.g., because of their clinical complexity) have protocols based on different guidelines that are not standardised across the health care system. Secondly, multidisciplinary cancer care displays different levels of development, so that patients in one hospital may be referred to a specific department, but not to the tumour board, in another, the point here being the absence of pre-specified criteria for referral among levels of care. Thus, some decisions are made without the scientific consensus of an MDT. Finally, a cancer team might change the original treatment plan for a patient referred from a lower level hospital. This was a concern voiced by several clinicians, since decisions sometimes tend to differ widely, causing confusion and lack of trust in the patient. This perception was not shared by health professionals who work for cancer networks.

"When a surgeon comes along saying that he has operated on a given patient without the consensus of the committee but -according to him- this course of action was 'in line with the evidence'... this is unacceptable. It's an issue to be addressed by the respective cancer plans. When guidelines are reviewed, this should be the starting point, i.e., the tumour committee report should be seen before the surgical report." (Radiation oncologist)

#### The role of the hospital executive board

Most health professionals believed that, while they had not been hampered by the hospital executive board, neither had they been specifically supported to better organise clinical pathways and MDT activity. In their view, the main problem was that MDT work time was not recognised as a health care activity (or "real work" to quote them). Half of those interviewed felt that hospital managers knew little about their tasks, goals, level of involvement and management problems. These professionals identified two clear priorities for hospital executive boards, namely: to protect multidisciplinary meetings and work time; and to promote new professional roles, such as nurse case-managers or administrative support. Those with management responsibilities stated that cancer teams were not reflected in the organisational chart but were very important in terms of quality of care, and more innovative and responsive insofar as health care organisation achievement was concerned.

"If you tell management that you have to attend a committee meeting, they view it as something that is all well and good but nevertheless ancillary, and so not meriting consideration as part of the daily work load. Yet such attendance should be accorded health care and scientific value, i.e., so many hours correspond to committee work, which is equal to time spent seeing patients in a medical practice." (Colorectal surgeon)

"Your personal efforts are not appreciated, regardless of whether you've taken part in drawing up a protocol or whether you've devoted one day or three weeks to the job... And, as no stress is laid on the importance of teamwork, there are pockets of resistance that don't change". (Radiologist)

#### Outcome assessment

The main goal of any multidisciplinary cancer team is to enhance the effectiveness of diagnosis and treatment of a specific disease. Assessment of the MDTs that had been put in place revealed relevant differences among the views held by the professionals themselves. To most of them, the ultimate consequence of the efforts of some hospital units or specialised professionals that regularly collect clinical data was evaluation or a study aimed at assessing MDT outcomes. Others, in contrast, described process evaluation involving initial inter-departmental consensus on indicators, development of a specific data-collection methodology, and periodic analysis of results using a shared database. Above all, this situation defines different approaches to the possibility of taking clinical outcomes and process indicators, and linking these to actions aimed at improving cancer care. There were two recurring arguments associated with possible ways of achieving organisational change: the first centred on the key role to be played by the health care service in reaching a technical definition of and agreement on a minimum set of indicators for the entire hospital system and a proposed level of transparency vis-à-vis outcomes; the second addressed the pervasive "culture of efficiency" currently prevailing in hospital departments, insisting on the need to limit its influence and instead give increased relevance to clinical and process indicators. An experience that has had remarkable success in various health care regions and has served as the basis for the evaluation of each MDT, is the implementation of a fast-track, colorectal, breast and lung cancer diagnosis and treatment programme, a *driving force *in promoting integration among services and MDTs. Its implementation has shown the key role that health care policy could play in enhancing the organisation of cancer care.

"The problem is that each specialisation has developed its own indicators of toxicity, clinical results, etc. There should be at least one database in which the team's outcomes are reflected. This is something that the hospital ought to demand. We could then say in real time, 'this, or that, is what's happening in prostate cancer'." (Medical oncologist)

"Yesterday I saw 37 patients: I can't devote myself to recording that much information in the database without any support... It's difficult for everyone's survival to be ascertained under such conditions. We tend to move within a 'dead' database context, that's to say, we get together at the end of the year to see how things have gone..." (Colorectal surgeon)

#### Recording and documenting of clinical decisions in an MDT setting

The more formalised MDTs become, the more important easy access to and transparency of decisions and the rationale behind them are. The reason for this is that recording decisions reflects the outcome of consensus building and the value that professionals attribute to their work. Half of all clinicians interviewed stated that they noted their decisions on the electronic clinical record. Not only does such action clearly define the end-point of the decision-making process, it also renders it more transparent, something that, in turn, generates a positive perception of the entire hospital environment. In contrast, there are many cases where team decisions do not extend beyond the strict limits of the tumour board, as shown by the first comment in Excerpt 5. The major weaknesses in recording clinical decisions stem from the lack of standardisation achieved in tumour-board Minute-taking, due to absence of common forms, failure to identify clear recording responsibilities and, very often, lack of administrative support. What this tends to mean is that only the decisions affecting of-protocol patients are recorded, thus hindering the possibility of establishing a reference database for a specific cancer. One last very important aspect for any team is the recording of decisions made in those cases where there is no consensus. Though infrequent, this situation is thought to play a relevant role in terms of medico-legal implications.

"In one of the hospital teams, there are professionals who find it difficult to accept consensus-based decisions. Accordingly, we consider it appropriate that, in addition to the decision being recorded in the digital clinical history, a file should be circulated to all team members so that decisions taken with respect to all patients are 'known' to them..." (Medical oncologist)

"We keep a number of formal records, I mean to say that there are several specialists who record details of patients in their files... but there is no single overall record." (Nurse case manager)

"There is an element of administration (which should be the task of a secretary) entailed in the drafting and signing of Minutes. This is generally performed by a physician, but if he's absent for any reason, then no-one does it. It's always the same old story: it's all a matter of personal involvement." (Pathologist)

## Discussion

The reference to Jean-Baptiste Lamarck (1744-1829) in the title of this article ("the function creates the organ") is a description that is both accurate and useful for understanding the evolution of multidisciplinary cancer care in the Spanish health system. In a manner similar to the apocryphal example of Lamarck's giraffe, which craned its neck to match the height of the trees, the development of cancer teams results from adaptation to the immediate hospital environment accompanied by a lack of policy orientation. While the law lays down that all hospitals are to have cancer boards for the most prevalent diseases, no specific aims, organisational requirements or performance assessment standards have been prescribed.

There is a valuable lesson to be learnt in the path taken by the UK National Health Service. The publication of the Calman-Hine [12] report in 1995 highlighted the importance of a successful institutional framework for cancer services. As Haward [13,14] pointed out, however, the effort to define their performance -- including multidisciplinary care-- in detail [15], without addressing the factors that were to facilitate the transition, resulted in slow, uneven change. The Spanish experience failed to develop this type of learning process. Our study therefore sought to identify the cultural and organisational dimensions that influence the incorporation of planned actions. This approach is reinforced by the EUROCARE-4 study, which identifies the organisational elements in the care process by the latter's ability to improve the survival and quality of life of cancer patients, as evidenced by the differences among European countries [16,17].

### Impact on decision making

Implementation of multidisciplinary care involves a redistribution of the responsibilities assumed by the respective professionals, with the aim of developing greater potential for enhancing their joint clinical effectiveness. It is a specific organisational answer to the complexity of cancer care, and enables new approaches to be taken and known problems, such as variability in clinical practice, to be tackled. In this connection, note should be taken of the overall strategy adopted by the National Breast and Ovarian Cancer Centre of Australia, which, along with several other authors [18-20], identifies multidisciplinary care with the standardisation of clinical practice in the health system. Most professionals interviewed by us regard MDT as the main tool for ensuring that the expertise of each discipline is involved in the clinical decision making process affecting any given patient. Furthermore, high levels of adherence to clinical protocols improve the efficiency of MD meetings by better discerning the transition from simple to complex case discussions.

Our study confirms previous research in underscoring the high degree to which the effectiveness of multidisciplinary interventions is dependent on the organisational context in which cancer care is delivered. Some technical aspects stressed are the need: for administrative support for team activity and organisation [8]; and for all decisions taken to be entered into the electronic clinical record, since failure to keep a record hinders application of such decisions to the patient, as shown by a study that targeted breast cancer teams in 2006 [21]. Moreover, a treatment-planning register can be helpful when it comes to assessing similar cases or auditing an MDT's performance [22]. Nevertheless, the key factor is communication among team members as a sign of professional team trust. This is the most relevant dimension to be discerned in the above-described models of integration of clinical care. The fact that decisions should be binding upon team members, that there is continued participation by specialists in the meetings, that the impact on the entire patient pathway is perceived as positive, that there is a role for clinical co-ordinators and nurse case managers, and that both residents and nurses participate in training, are indicators of the ability of the clinicians involved to abandon a sequential and relatively unco-ordinated model of cancer care and progress instead towards achieving a model of integrated care based on consensus decision-making.

Specialisation in a given area of cancer diagnosis and treatment facilitates communication among different specialisations and professionals, through using specialist knowledge and expertise as a departure point for addressing specific patients rather than for the performance of specific tasks. Experiences, such as reaching an agreement on a common gateway for patients with high-risk symptoms for cancer or protecting the time for multidisciplinary teamwork, can also be key groundwork for promoting effective team communication. Other researchers emphasise aspects of the importance of professional integration within cancer networks [23], or the improvement in mental wellbeing and professional satisfaction that comes with MDT development, as a result of lower anxiety and better feelings about personal performance [24,25]. Another approach is also the need to achieve consistent care from the standpoint of the cancer patient [26]. This was well illustrated by affording patients access to the MDT in the early stages of the diagnostic process, a way of preventing the possibility of initial treatment being administered without team discussion, and communicational fragmentation with the patient being increased.

### Strengths and limitations

This study has some strengths and limitations that must be taken into account when assessing its results. Insofar as its strengths are concerned, it should be noted that, rather than approaching MDTs from the standpoint of the specific structures which frame teamwork, we sought instead to understand MDTs from the standpoint of the capabilities of the professionals and teams themselves. This enabled us to obtain a better insight into the ways in which professionals interacted and the nature of the agreements and commitments reached within an MDT. The synthesis of our results in the form of three models of multidisciplinary cancer care to be found in Spain facilitates the transfer of such findings to SNHS hospitals. Indeed, as our study shows, multidisciplinary care displays significant variability in its methodology and degree of implementation among hospitals and regions, but not in the critical factors that have influenced its development. These models have been checked with the health professionals involved in the study.

A clear limitation of the study resides in the selection process, based on proposals put forward by the chairmen of scientific societies of medical and radiotherapy oncology and the heads of regional cancer plans, which could have biased our selection of professionals towards those with sensitivity to multidisciplinary care and organisational change *per se*. The selection criteria vis-à-vis the different profiles, plus the fact that major university teaching hospitals were involved in the study, were intended to minimise this limitation. Moreover, our interpretation of the findings and the model proposed here were discussed with different specialists, hospitals and regions.

As with all qualitative studies, there was not a large number of participants. Our research focused on the views of key informants, thereby implicitly ruling out the possibility of capturing all the experiences and best practices that might exist in the health system. Lastly, mention should be made of the fact that one third of all interviewees belonged to the field of breast cancer, a disease that frequently becomes a model for others.

## Conclusions

This is the first qualitative study of multidisciplinary cancer care in southern Europe. The delay in MDT implementation poses the need for health policy not only to acknowledge and promote it, but also to provide quality standards. In addition, there is a clear need to respect and promote good practices existing in the health care system. In this regard, this study may help understand how professionals conceptualise this approach, which is relevant when interest lies in developing more comprehensive care by placing multidisciplinary care at the core of cancer department, as stated in Spain's official cancer strategy. Moreover, metaphors play a key role in the way professionals imagine and explain teamwork in cancer care (table 4).

**Table 4:** Research metaphors

The "black box"	This metaphor is often used by health professionals outside MD meetings because of little knowledge of their internal functioning.
"The Lone Ranger"	The "Lone Ranger doing 'clinical justice' is outdated but we still have many 'Lone Rangers' riding in our health system", says one interviewee. Lone Rangers, in this context, are clinicians who unilaterally assume the management of cancer processes.

"Orchestra" vs. "Big Band Jazz"	In the case of the orchestra, a multidisciplinary team requires a "baton to lead it", a few "first violins to give the health care symphony order and structure" and several "instruments" which may stand out to a greater or lesser degree but must nevertheless all play in harmony so that the ensemble sounds good as a whole. To this end, developing an internal organisation based on commonly shared rules and roles is a crucial factor. Other professionals view "Big Band Jazz" as a more appropriate metaphor. They understand the functioning of the multidisciplinary team in a much less rational and formalised way, a human group in which improvisation and voluntary actions play a key role, with individual creativity as an essential component for ensuring that the process has a good outcome.

"Partitions and walls"	Professionals refer to the different metaphorical thickness of the partitions and walls to explain the mental distances that often separate them.

"Main actors, supporting (secondary) actors, and guests artists"	The feeling of playing specific roles in teams varies among professionals. Some of them express their involvement in terms of being main actors, and others as supporting actors or guests artists who attend the meeting only because they are invited.

The "snowball"	The large volume of visits entailed in long-term follow-up of cancer survivors, equivalent to one third of the time of activity for some professionals, leads them to refer to this process as a "snowball". In fact, one physician interviewed stated that, "you almost marry a patient with cancer".

In "no man's land" or "trapped between the two health systems"	These expressions are used in cases where good practices for taking care of cancer survivors are seen to be lacking, and the current intervention model is ineffective. Primary and specialised care are organised and conceived without identifying specific needs and consistent responsibilities vis-à-vis cancer survivors.

MDT development often entails a process of decentralisation inside hospitals, which may involve some redistribution of power. This is an adaptive challenge for hospital managers in terms of clinical governance, i.e., making structures more permeable to organisation of expertise without losing efficiency in the management of shared resources. A culture of evaluation of clinical and process outcomes should emerge, aimed at directing and justifying organisational innovation so as to achieve the best possible performance in terms of care of cancer patients. Multidisciplinary care occurs simultaneously with rapid changes in treatment and use of clinical practice guidelines, all of which makes it more difficult to identify its specific advantages [27]. This is why health policy plays an important role in promoting an organisational approach that changes the way in which professionals develop their clinical practice, a key issue in a disease such as cancer, characterised by its clinical complexity, involvement of different clinical specialists and need to face the new challenge of managing patient preferences.

## List of abreviations

MDT: Multidisciplinary Team; SNHS: Spanish National Health System

## Competing interests

The authors declare that they have no competing interests.

## Ethical approval

Not required.

## Authors' contributions

JMB had the initial idea for this study. JMB and JP designed the study and drafted the research proposal. JP conducted pilot interviews, while JMB provided guidance and critical review of this information and helped with the review of the literature. JP undertook the main fieldwork for the study, interviewed, coded, charted and analysed the data for this paper, which was scrutinised and discussed by JMB. JP and JMB interpreted the results and wrote the first draft and final version of this article. JP and JMB read and approved the final manuscript.

## Pre-publication history

The pre-publication history for this paper can be accessed here:

http://www.biomedcentral.com/1471-2458/11/141/prepub
